# HTLV-1 and HTLV-2 Infection Among Warao Indigenous Refugees in the Brazilian Amazon: Challenges for Public Health in Times of Increasing Migration

**DOI:** 10.3389/fpubh.2022.833169

**Published:** 2022-02-11

**Authors:** Isabella Nogueira Abreu, Felipe Teixeira Lopes, Carlos Neandro Cordeiro Lima, Alexandre do Nascimento Barbosa, Lehi Rodrigues de Oliveira, Mayumi Aragão Fujishima, Felipe Bonfim Freitas, Mike Barbosa dos Santos, Vitor Nina de Lima, Izaura M. V. Cayres-Vallinoto, Socorro Castelo-Branco, Hilton P. da Silva, Antonio Carlos R. Vallinoto

**Affiliations:** ^1^Laboratório de Virologia, Instituto de Ciências Biológicas, Universidade Federal do Pará, Belém, Brazil; ^2^Faculdade de Medicina, Instituto de Ciências da Saúde, Universidade Federal do Pará, Belém, Brazil; ^3^Seção de Virologia, Instituto Evandro Chagas, Ananindeua, Brazil; ^4^Secretaria de Saúde do Município de Belém (SESMA), Belém, Brazil; ^5^Programa de Pós-Graduação em Antropologia e Programa de Pós-Graduação em Saúde, Ambiente e Sociedade na Amazônia, Universidade Federal do Pará, Belém, Brazil

**Keywords:** migration, Warao, Venezuelans, Amazon, public health

## Abstract

**Introduction:**

Human T-lymphotropic virus (HTLV) infection is endemic in indigenous populations of the Americas. We describe herein the prevalence of HTLV-1 and HTLV-2 infection among Warao indigenous refugees from Venezuela living in Belém, Pará, Brazil.

**Methods:**

In total, 101 individuals of both sexes (43 men and 58 women) between 18 and 77 years of age were investigated. Blood samples were collected and separated into plasma and leukocytes. Serological screening was performed using an enzyme-linked immunosorbent assay (ELISA; Murex HTLV-I+II, DiaSorin, Dartford, UK), and seropositive samples were submitted to proviral DNA extraction followed by real-time polymerase chain reaction (qPCR). A nested PCR of the *env* region (630 bp) followed by enzymatic digestion with *Xho*I was performed to identify the molecular subtype of HTLV-2, in addition to sequencing analysis of the 5'LTR-I and 5′-LTR-II regions.

**Results:**

Of the 101 individuals analyzed, 3 (3.0%) were seropositive. Molecular analysis of the *pol* and *tax* genes confirmed the HTLV-1 infection in a 55-year-old woman and HTLV-2 infection in a man (68 years old) and a woman (23 years old). HTLV-2 strains were defined by enzymatic digestion as belonging to the HTLV-2b subtype. The sequencing of the 5′LTR regions confirmed the presence of subtype 2b and identified HTLV-1 as belonging to subtype 1A (Cosmopolitan) and the Transcontinental subgroup. Among the infected patients, it was possible to conduct medical interviews with two individuals after delivery of the result. One patient with HTLV-2 reported symptoms such as joint pain, foot swelling, frequent headache, dizziness and lower back pain. The HTLV-1-positive woman was diagnosed with a tumor, dementia, urinary incontinence, felt body pain, and had spots on her body. The presence of the HTLV-2b subtype highlights the prevalence of this molecular variant among indigenous South Americans, as well as the presence of HTLV-1 Transcontinental, which has a worldwide distribution.

**Conclusion:**

These results reveal a high prevalence of HTLV-1/2 infection among Warao immigrants, suggesting migratory flow as a virus spread mechanism among human populations and alert public authorities to the need to create epidemiological surveillance programs, public social and health policies aimed at welcoming immigrants in the Brazilian territory.

## Introduction

Human T-lymphotropic viruses 1 and 2 (HTLV-1 and HTLV-2) are members of the *Retroviridae* family that infect humans and have numerous similarities in biological and molecular properties (1).

HTLV-1 infection has endemic characteristics in well-defined geographic regions such as southern Japan, the Caribbean, and Australia (2, 3), where the infection is associated with adult T cell leukemia (ATL), uveitis, and a chronic neurological disease known as HTLV-1-associated myelopathy (HAM). HTLV-1 infection is present with varying frequencies among American indigenous peoples, and its origin is attributed to the migratory flow of ancestors or, more recently, to the trafficking of African slaves during the colonial period (4).

HTLV-2 infection has an endemic distribution in Amerindian populations (5–7). In North America, HTLV-2 is present in the Navajo and Pueblo Indians of New Mexico (8, 9) and among the Seminoles in Florida (10). In Central America, the infection is endemic among the Guaymi of Panama (11–13) and Maya of Mexico (14). In South America, the infection has been described in the Wayu, Guahibo, Tunebo, and Orinoco of Colombia (15–19), the Toba, Mataco, and Mapuche of Argentina (20–22), and Gran Chaco of Paraguay (22). In Brazil, the presence of HTLV-2 is described as hyperendemic in several ethnic groups, with emphasis on the Kayapó people, where the prevalence reaches percentages above 30% in some villages, with the endemicity being associated with the spread of the virus through mother-child (by breastfeeding) and sexual transmission routes (6, 23).

Molecular studies demonstrate the circulation of three HTLV-2 molecular subtypes among these different indigenous peoples of the Americas, with HTLV-2a and HTLV-2b (24) being prevalent among peoples of North, Central and South America (13, 14, 19, 22, 25–27) and the HTLV-2c subtype restricted to indigenous peoples of the Brazilian Amazon (6, 7).

The origin of HTLV-2 infection among the native peoples of the Americas has been attributed mainly to the migratory flow of the Asian ancestors of the current Amerindian peoples who ~30,000 years ago migrated toward North America *via* the Bering Strait, with a subsequent descent toward Central and South America (28, 29). This migratory flow would have introduced subtypes 2a and 2b among the current Amerindian peoples, with the exception of the native peoples of Brazil, whose 2c variant seems to have evolved independently after the differentiated migration of ancestral peoples toward the Amazon (7, 30).

Past and current human migratory flows are important mechanisms for the dissemination, emergence and re-emergence of infectious agents in different geographic areas (28), having a marked role in the origin and spread of HTLV-1 and HTLV-2 from the African continent toward Europe, Asia and the Americas (4, 29, 31). Currently, social, political and economic crises have motivated intense migratory movements and asylum requests in Latin American countries, which certainly favors the dispersion and emergence of infectious agents to new geographic areas. In this sense, in the present study, we investigated the occurrence of HTLV-1/2 infection and its subtypes in Venezuelan immigrants of the Warao ethnic group living as refugees in the city of Belém, one of the largest metropolises in the Brazilian Amazon, to alert public authorities to the need to create epidemiological surveillance programs, public social and health policies aimed at welcoming immigrants, and the adequate prevention of the spread of HTLV-1/2 in the Brazilian territory.

## Methods

### Warao Population

**Figure 1:**
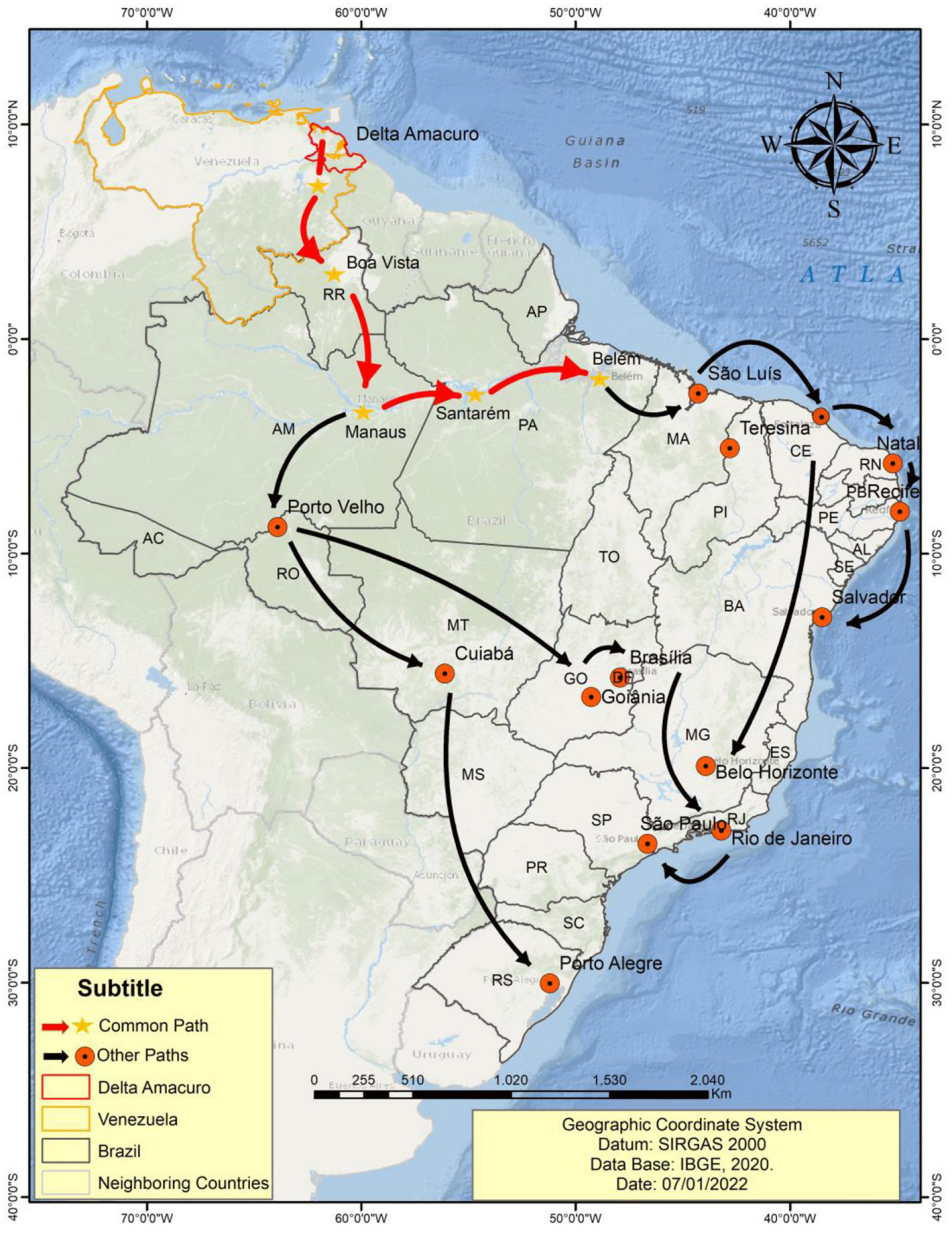
Map representing the migratory flow of the Warao people from Venezuela to Belém (State of Pará) and other Brazilian capitals.

The Warao people are the second most populous indigenous group in Venezuela. Originally, they were located predominantly in the Caribbean region of the Orinoco River Delta, in hundreds of communities dispersed in rural, riverside, and coastal areas and in several cities in the state of Delta Amacuro and regions of the states of Monagas and Sucre, with archaeological records of their presence in the area since 8 thousand years ago. The economic and ecological pressures on their territories have been ongoing for several decades, but they have become more acute in the last 10 years, leading the Warao people to seek refuge in other bordering countries (32, 33). They started to migrate to Brazil in mid-2014, with migration intensifying with the increase in the Venezuelan crisis in 2016, reaching the city of Belém (Pará, Brazil), as well as other Bazilian capitals the following years (Figure 1). The Warao migrated mainly in search of a better quality of life and were motivated by the political, economic and humanitarian crisis in their country. In Brazil, family groups ultimately reside in public or self-managed shelters or even live in street conditions and extreme poverty, often being exposed to drug trafficking and prostitution (34, 35).

In Venezuela, the Warao had fishing, crafts and the collection of forest products as their main source of income. In their culture, women are responsible for taking care of the home, raising children and producing handicrafts sold in urban areas. In Brazil, they are also responsible for collecting money at traffic signals. Men, on the other hand, are responsible for feeding the family and building houses (33).

Culturally, they try to maintain the role assignments between men and women when they are refugees in other countries. However, due to differences in territories, they ultimately try other means of support. In some places, due to factors such as prejudice, xenophobia and the absence of public policies, indigenous Venezuelans are unable to enter the labor market and become involved in crime and prostitution (34–36).

### Ethical Aspects and Sample Collection

A total of 101 whole-blood samples were obtained from individuals self-declared as belonging to the Warao ethnic group, all of whom were from Venezuela and were living in a refugee situation in the metropolitan region of Belém, Pará, Brazilian Amazon. The project's objectives were presented to the communities in Spanish and Warao, after which prior consent was obtained from the leaders to carry out the research. All volunteers were interviewed with the help of an interpreter, and after agreeing to participate in the research, they signed a free and informed consent term. The project followed the guidelines of the Declaration of Helsinki and was approved by the Ethics Committee for Research on Human Beings of the Health Sciences Institute of the Federal University of Pará (CAAE: 31800720.1.0000.0018).

Collections were carried out from September 2020 to January 2021. From all participants, 4.5 mL of venous blood was collected in a vacuum tube containing EDTA (ethylenediamine tetraacetic acid) as an anticoagulant. Then, the samples were transported to the Virology Laboratory of the Federal University of Pará in an insulated box. The samples were submitted to plasma and leukocyte separation by means of centrifugation at 8,000 rpm for 15 min, followed by storage at −20°C until analysis.

### Serological Screening

The samples were submitted to serological screening for the detection of anti-HTLV-1 and anti-HTLV-2 antibodies using an enzyme-linked immunosorbent assay (ELISA; Murex HTLV-I+II, DiaSorin, Dartford, UK).

### Real-Time PCR

ELISA-positive samples were subjected to DNA extraction using a QIAamp DNA Mini and Blood Mini Handbook Kit (QIAGEN, Hilden, Germany).

qPCR was used to confirm and differentiate HTLV-1 and HTLV-2 infection. In each sample, the human albumin gene was used as an endogenous control, and the viral gene regions pol (186 bp) and tax (75 bp) (37) were amplified.

The samples were tested in triplicate using Applied Biosystems Step One Plus Real-Time PCR equipment. For each reaction, 12.5 μL of TaqMan Universal PCR Master Mix (2X), 6.0 μL of ultrapure water (H_2_O), 0.5 μL of each primer, 0.5 μL of each probe and 5.0 μL of DNA were used, resulting in a total volume of 25 μL. The following temperature cycles were used: 95°C for 10 min, followed by 45 cycles of 95°C for 15 s and 60°C for binding of primers and probes for 1 min.

The primers used were 5′-CCCTACAATCCAACCAGCTCAG-3′ (HTLV-1F), 5′-GTGGTGAAGCTGCCATCGGGTTTT-3′ (HTLV-1R), 5′-CGATTGTGTACAGGCCGATTG-3′(HTLV-2F), 5′-CAGGAGGGCATGTCGATGTAG-3′ (HTLV-2R), 5′-GCTGTCATCTCTTGTGGGCTGT-3′ (Albumin F), and 5′-AAACTCATGGGAGCTGCTGGTT-3′ (Albumin R), and the probe sequences were FAM-5′-CTTTACTGACAAACCCGACCTACCCATGGA-3′-MGB (HTLV-1), FAM-5′-TGTCCCGTCTCAGGTGGTCTATGTTCCA-3′-MGB (HLTV-2) and FAM-5′-CCTGTCATGCCCACACAAATCTC-3′-MGB (Albumin) (37).

### Restriction Fragment Length Polymorphism

Samples confirmed by qPCR as HTLV-2 positive were subjected to molecular subtype identification through a nested PCR of the env region (630 bp), followed by enzymatic digestion using the restriction enzyme XhoI, which generates two fragments (430 and 180 bp). The first reaction consisted of 29.0 μL of water (H2O), 5.0 μL of buffer (10X), 1.5 μL of MgCl2 (50 mM), 0.5 μL of primer (E2) 5′CTGCAGAAGCTAGCAGGTCTA-3′ (20 pmol), 0.5 μL primer (E5) 5′-AGCCAAGTGTCCCTTCGACTA-3′ (20 pmol), 8.0 μL of dNTPs (1.25 mM), 0.5 μL of Taq (1 U/μL), and 5.0 μL of DNA.

The second reaction followed the same protocol, using the primers (E1) 5′-CTGCAACAACTCCATTATCCT-3′ and (E2) 5′CTGCAGAAGCTAGCAGGTCTA-3′ and 5.0 μL of DNA from the first reaction (24). Both reactions employed the same temperature cycles: 94°C for 3 min and 35 cycles of 94°C for 40 s for denaturation, 53°C for 30 s for annealing primers, and 72°C for 40 s for extension.

For enzymatic digestion with XhoI, 7.3 μL of water (H_2_O), 2.0 μL of RE Buffer (10X), 0.2 μL of BSA, 10.0 μL of DNA from the second PCR and 0.5 μL of the XhoI enzyme were incubated for 4 h at 37°C. The products were submitted to 3% agarose gel electrophoresis at a voltage of 100 V for 40 min and visualized with ethidium bromide staining.

### Nested PCR of the 5′ Long Terminal Repeat Region

The sample confirmed for HTLV-1 was subjected to amplification of the 5′LTR-1 region, with the aim of characterizing the molecular subtype. First, 11.45 μL of ultrapure water (H_2_O), 1.25 μL of buffer (10X), 3.0 μL of MgCl_2_ (50 mM), 6.0 μL of dNTPs (10 mM), 0.5 μL of each primer (20 pmol), 0.3 μL of Taq (1 U/μL) and 2.0 μL of DNA were combined. The following HTLV-1 primer sequences were used: (LTR-I.01) 5′-TGACAATGACCATGAGCCCCAA-3′, (LTR-I.02) 5′-CGCGGAATAGGGCTAGCGCT-3′, (LTR-I.03) 5′-GGCTTAGAGCCTCCCAGTGA-3′, and (LTR-I.04) 5′-GCTAGGGAATAAAGGGGCGC-3′. For HTLV-2, (F-IILTR) 5′-TCGCGATGACAATGGCGACTAGCCTC-3′, (Long-Gag) 5′-GGGGGCTTTGGGTATTGGAGTTGGG-3′, (Mo16) 5′-GCCTCCCAAGCCAGCCAC-3′, and (MSW-Gag) 5′- GGGAAAAGCCCGTGGATTTGCCCCAT-3′ were used. The following temperature cycle was used: 94°C for 5 min; 35 cycles at 94°C for 40 s, 62°C for 30 s and 72°C for 40 s; ending at 72°C for 10 min.

### DNA Sequencing and Phylogenetic Analysis

After purification of the PCR product (5'LTR region), the amplified DNA was sequenced using the Sanger method with a BigDye Terminator v3.1 Cycle Sequencing Kit (Thermo Fisher, Waltham, MA, USA) using Genetic Analyzer 3130xl equipment (Applied Biosystems) (38).

Sequence alignment of 430 bp for HTLV-1 and 534 bp for HTLV-2 of the 5′LTR region was performed using the Clustal W program implemented in BioEdit software v7.1.9 (39). The phylogenetic relationships between the sequences described in the present study (HTLV-1 - BRPA_31564 and HTLV-2 – BRPA_31238) and those available in GenBank for HTLV-1 (BRPA; 146, BRPA; 180, CQ443748; BS130, EU108721; CA422, CQ443755; K344, DQ005558; HTLV06, CQ443757; K535, D1005565; HTLV24, M37299; H5, U12804; Algerian, U12806; Pr52 Moroccan, U12805; OD Mauritanian, and L76310; pyg19, JX501) and for HTLV-2 (AF306735; BrBel, AF306734; BrBel, AF306725; BrBel, AF306726; BrBel, U10253; Br, AF306733; BrPa Kararao, L42509; Br Kayapo, L42508; Br Kayapo, AF306731, BrPa Kararao, AF306730; BrPa Tira; BrPa Kararao, AF306724; BrBel, AF306728; BrPa Gorotire, AF306727; BrPa Gorotire, AF306729; BrPa Tiriyo, L42507; Ghana, L42510; Mex, U10257; USA, Z46838; Afr, U10256; USA, U1060; USA, M10060, U10252; USA, U10258; Nor, Y14364; Bambuti Afr, HTLVII_Y13051; Gab, L207; USA, Z46888; Afr, U10263; USA, U12794; Wayu Col, U10260; USA, U10264; USA, U10261; USA, L11456; Guay mi, U12792; Wayu Col, X89270; Ita, U10255; ItA, L77241; Esp, L77237; Esp, U10254; Ita, U10259; USA, L77244; Esp, L77238, Esp, U10265, Esp, L77236; Esp, L77235; Esp, and U10266; Esp) were inferred by the Bayesian method in the program MrBayes v3.2.7 (40), using the replacement Hasegawa-Kishino-Yano (HKY) models for HTLV-1 and the GTR+G model for HTLV-2 in the program JmodelTest v2.1.10 (41). The statistical reliability of the Bayesian tree was evaluated using 1,000 bootstrap replicates and visualized using the FigTree v1.4.4 program (42).

## Results

### Serological and Molecular Analysis

Among the 101 samples analyzed, the mean age of the participants was 36 years, ranging between 18 and 77 years. Three individuals (3.0%) tested positive by ELISA. Afterward, the infection was confirmed by characterizing of the viral type in the samples by means of qPCR analysis for the pol and tax genes.

HTLV-1 infection was confirmed in a 55-year-old woman (1%), and HTLV-2 was present in a man (68 years old) and in a woman (23 years old) (2.0%). Of the three positive samples, it was possible to amplify the 5′LTR regions in only two for subsequent sequencing and phylogenetic analysis.

**Figure 2:**
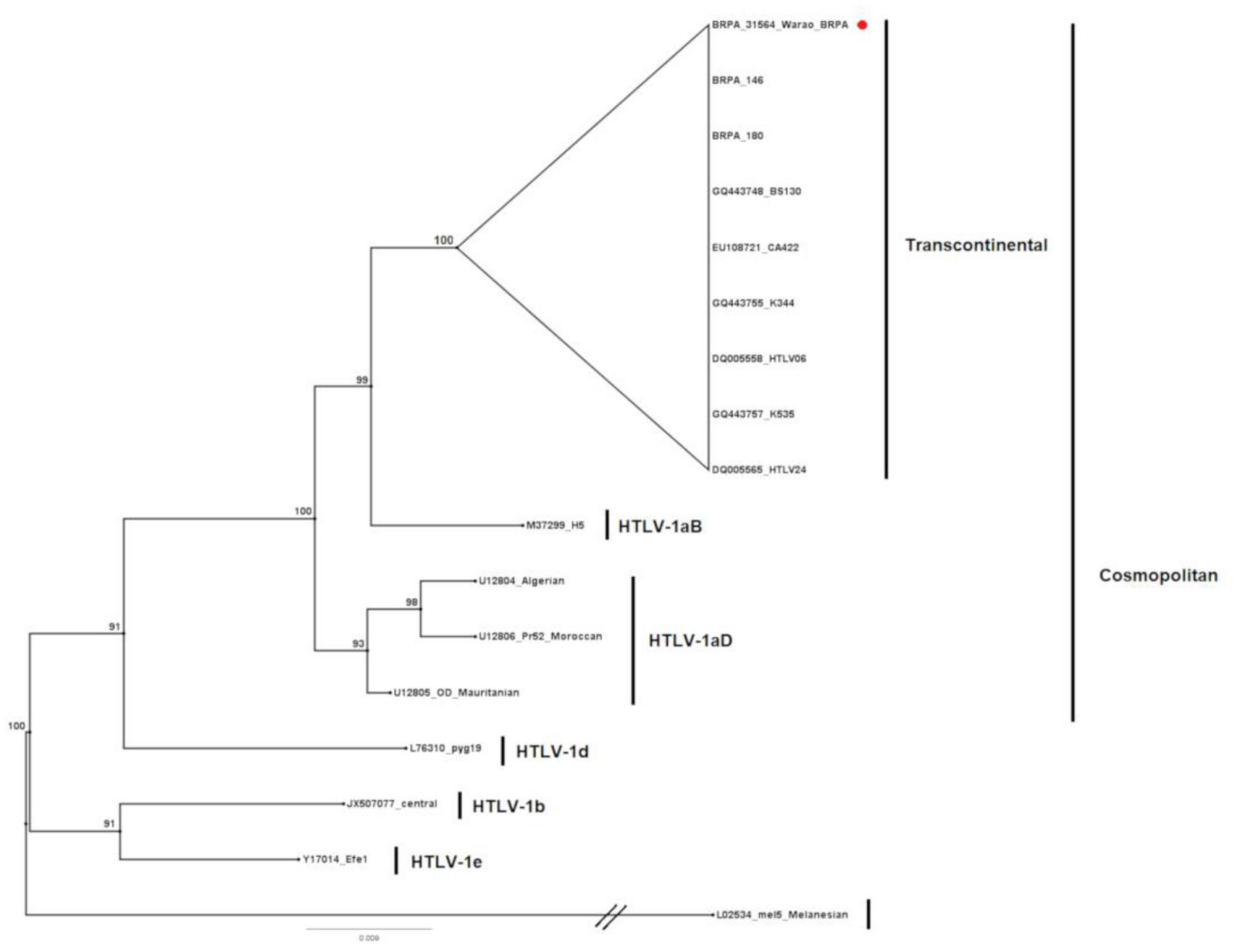
Rooted phylogenetic tree showing the relationships among the HTLV-1 samples available in GenBank and the one described in the present study (BRPA_31564_Warao). The tree was constructed using the Bayesian method after aligning 430 nucleotides from the 5′LTR-1 region. The statistical sustainability test (bootstrapping) was applied using 1,000 replicas of the sequence bank.

The sample characterized as HTLV-1 positive showed, in the phylogenetic tree, 100% grouping with samples of the Cosmopolitan subtype (HTLV-1a), Transcontinental subgroup (A) (Figure 2).

**Figure 3:**
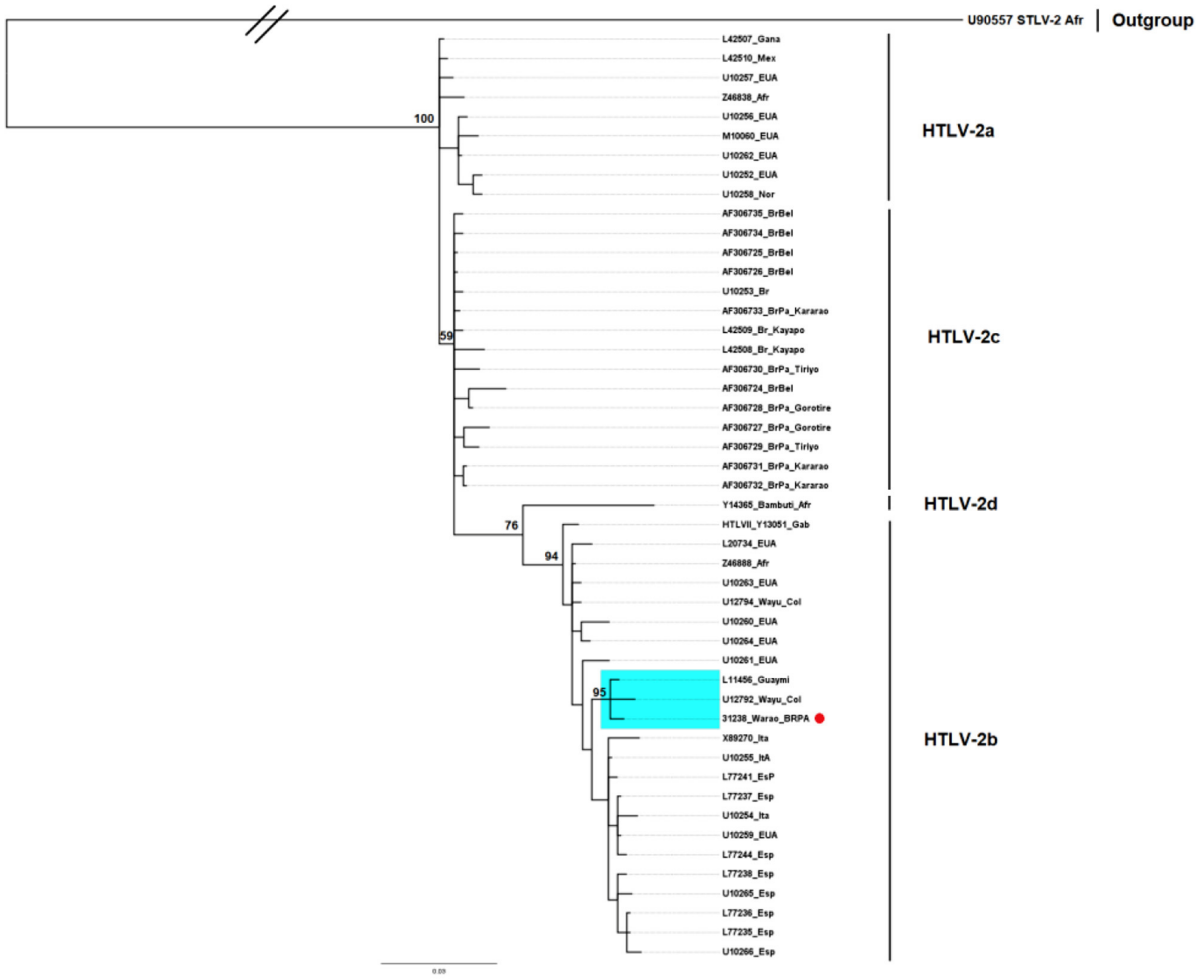
Rooted phylogenetic tree showing the relationships among the HTLV-2 samples available in GenBank and the one described in the present study (BRPA_31238_Warao). The tree was constructed using the Bayesian method after aligning 534 nucleotides of the 5′LTR-2 region. The statistical sustainability test (bootstrapping) was applied using 1,000 replicas of the sequence bank.

The HTLV-2 detected in one of the samples grouped in the phylogenetic tree, with 76% support, with samples belonging to the HTLV-2b subtype (Figure 3), forming a clade with 95% identity with strains isolated from the Guaymi (L11456) and Wayu (U12792) indigenous communities from Colombia. Although it was not possible to perform amplification and sequencing of the 5′LTR region in one of the HTLV-2-infected samples, nested PCR of the env region followed by enzymatic digestion with XhoI confirmed that the sample also belonged to subtype 2b.

### Clinical-Epidemiological Characterization of HTLV-1/2-Seropositive Individuals

As part of the Screening and Counseling Program for People Living with HTLV implemented by the UFPA Virology Laboratory, the results of the serological screening and molecular confirmation were delivered to all participants.

The three individuals positive for the infection were evaluated by a physician from the Belém Municipal Health Department for individual monitoring by the Unified Health System (SUS), at which time a consultation and interview were carried out to obtain information on risk factors and signs/symptoms associated with HTLV infection (Table 1).

**Table 1:** Epidemiological characteristics of HTLV-positive individuals belonging to the Warao ethnic group residing in the city of Belém, Pará, Brazil.

**Individual**	**Age**	**Sex**	**HTLV**	**Partners**	**Sexual**	**Sex**	**Age of first**	**Condom**	**Piercing**	**Tattoo**	**Received**	**Was breastfed**
				**per week**	**abuse**	**for**	**sexual**	**use**			**blood**	**as a**
					**report**	**money**	**intercourse**				**transfusion**	**child**
31,235	68	Male	2	1	No	No	20	No	No	Yes	No	Yes
31,564	55	Female	1	0	RA	RA	NA	No	No	No	NA	Yes

*RA, refused to answer; NA, could not answer*.

#### Individual #31564 (HTLV-1)

The individual is female, 55 years old, and single; has lived in Brazil for 4 years; is illiterate and unemployed; and earns less than half the national minimum wage per month (U$200). She did not have tattoos or piercing, reported not knowing about receiving blood transfusions, and reported having 25 children who were breastfed for more than 6 months. She was breastfed as a child. She refused to answer whether she had had sex with a sex worker during her lifetime or if she had been sexually abused. She stated that she did not use condoms during sexual intercourse and did not remember the age of first sexual intercourse.

The patient had a central nervous system tumor identified by CT at the health service, reported going to the bathroom more frequently than usual, felt pain in her body, was progressing to dementia, and had spots on her arm.

#### Individual #31235 (HTLV-2)

The individual was male and 68 years old, lived together with his partner and had resided in Brazil for 3 years. He could read and write Spanish, but he had incomplete basic education, was unemployed, received emergency aid from the government, and earned ~1 national minimum wage per month. He reported having been a smoker but had quit a long time ago. He did not drink alcohol, had a tattoo located on his arm, reported that he had never received a blood transfusion, had never used illegal drugs, was breastfed as a child, had his first sexual intercourse at age 20, had a steady sexual partner, did not use a condom during sexual relations, had never had sex with a sex worker and reported never having been sexually abused.

During the clinical evaluation, he reported having dizziness, frequent headache, joint pain, lower back pain and a swollen foot with spots for more than 1 year.

#### Individual #31238 (HTLV-2)

The individual was female and 23 years old, had lived in Brazil for 1 year, lived with her partner, knew how to read and write in Spanish, had incomplete basic education, and was unemployed with a family income between half and one national minimum wage per month.

Upon delivery of the result and interview with the physician for information about the presence of signs and symptoms, the patient refused to receive the result.

## Discussion

In the present study, we identified the presence of HTLV-1 and HTLV-2 infections in Warao immigrant refugees in Belém, capital of the State of Pará. There are few studies on the prevalence of HTLV-1/2 infection in Venezuela, with emphasis by León et al. (43) on blood donors, in which an occurrence of 0.2% was detected, by Márquez et al. (44) on patients seen at a health care unit (0.58%) and on indigenous populations, in which the prevalence of HTLV-2 subtype b infection was described as 61% in the Yaruro and Guahibo populations (45), which once again reinforces the presence of high frequencies of HTLV-2 infection in South American indigenous populations.

The first description of HTLV-2 in Venezuelan indigenous peoples occurred in 1993 in the Pumé ethnic group, with 5% of the samples being positive for HTLV-2. Perez et al. (46) found this prevalence when investigating two villages that had greater contact with non-indigenous individuals and four more isolated villages, describing the presence of the infection as something restricted to individuals who maintained contact with outsiders, suggesting a non-indigenous origin of the virus. The 3% prevalence observed in our study is close to that described for HTLV-2 among the Pumé, especially due to the character of a non-isolated population that the Warao present in the current context.

On the other hand, the description of the presence of HTLV-1 in the present study diverges from the three previous studies that investigated the seroprevalence for HTLV-1/2 in Venezuela (46–48). Notably, the presence of HTLV-1 subtype Cosmopolitan, Transcontinental subgroup, suggests the circulation of HTLV-1 among the Warao, perhaps as a result of a possible transmission from increased contact with non-indigenous individuals, as suggested by Vandamme et al. (4) regarding contacts with African-descendant populations living in the Americas.

Studies revealing a high prevalence of HIV-1 (9.55%) in Warao have also indicated a high probability of HTLV infection in this population since transmission routes and risk factors are common for these and other sexually transmitted infections (STIs) (48, 49).

The Warao refugee population in Brazil is looking for better living conditions, such as work and access to the education and health system. However, they ultimately live in precarious situations, living in makeshift shelters and facing situations of great social and economic vulnerability, commonly becoming victims of racism, other forms of discrimination and the absence of public policies that are culturally sensitive to their needs as indigenous people and refugees (35, 50). All of these aspects represent risk factors, with several reports of sexual abuse, drug use and sex in exchange for money (49).

In our study, the occurrence of HTLV-1 and HTLV-2 in two females is perhaps associated with a greater exposure of women to a situation of risk for STIs due to the living conditions faced by these indigenous communities, where women are subordinate to men, resulting in cases of sexual abuse, family violence and risky sexual practices (35).

To date, the results described here are unprecedented and reinforce the findings of the high prevalence of HTLV-2 in indigenous peoples, as already demonstrated by our group in populations from the Brazilian Amazon (6, 23, 30, 51). However, the presence of the HTLV-2b subtype represents a unique aspect in the epidemiological context of HTLV infection in the city of Belém, where the HTLV-2c subtype, frequent in the indigenous peoples of the region, prevails. Furthermore, the presence of subtype 2b was described only once in the capital Belém, and although it was not possible to clarify the entry route at that time, it was most likely the result of introduction by migratory flow (52). More recently, the same molecular subtype 2b was described among injection drug users in the State of Pará (53), reinforcing the need to implement molecular epidemiological surveillance to prevent the entry and spread of new viral strains in the population.

One of the hypotheses to explain the presence of HTLV-2b in the Warao people would be the proximity of their place of origin to the Guahibo people, in addition to the similar life habits (35). During the collections and interviews, it was possible to observe that there were, among the Warao residents in Belém, cases of union between individuals of different ethnicities and with non-indigenous individuals, suggesting possible alternative routes for the presence of the virus in the studied group. Another hypothesis would be that of sexual transmission through women who end up having a greater degree of exposure to harassment in large cities as a result of the sale of handicrafts or the act of asking for money at traffic signals. Similarly, for men, the risk is increased due to involvement with sex workers.

The sexual route being the main HTLV transmission route in the Warao population was supported by reports of not using condoms during sexual intercourse, increasing the susceptibility to STIs. Additionally, this hypothesis is reinforced by the precarious situation in which the Warao live, reporting lack of food, child malnutrition, and cases of pneumonia, tuberculosis, measles, coronavirus disease 2019 (COVID-19) and other diseases such as HIV and other STIs (35, 54, 55).

HTLV-1 infection is associated with neurological symptoms that can affect between 1 and 5% of those infected, especially affecting middle-aged women (56). In the clinical evaluation that HTLV-positive individuals underwent, it was possible to verify the occurrence of skin changes, body aches, urinary incontinence, dementia and diagnosis of a brain tumor in the 55-year-old patient with HTLV-1.

## Conclusion

Finally, our results reveal for the first time the circulation of HTLV-1 and HTLV-2 in the Warao ethnic group from Venezuela, who are refugees in the State of Pará (northern Brazil). The presence of the HTLV-2b subtype reinforces that this molecular variant is prevalent among the indigenous peoples of South America and highlights migratory flow as an important means of introduction and dispersion of HTLV in human populations, especially those in the Brazilian Amazon. Due to the economic, political, environmental and humanitarian crises that several countries in Latin America are experiencing, resulting in intense migratory flows that mainly affect the most vulnerable groups, national states need to plan and implement culturally sensitive public health policies to welcome and care for traditional peoples in their territories, as well as guarantee adequate epidemiological surveillance and health services (among others) for immigrants, following multilateral legal provisions and the principles of humanitarian assistance and international solidarity.

## Data Availability Statement

The datasets presented in this article are not readily available for reasons related to data confidentiality and participant privacy. Requests to access the datasets should be directed to Prof. Antonio Carlos R. Vallinoto ( vallinoto@ufpa.br).

## Ethics Statement

The studies involving human participants were reviewed and approved by Human Research Ethics Committee of the Health Sciences Institute of the Federal University of Pará (CAAE: 31800720.10000.0018). The patients/participants provided their written informed consent to participate in this study.

## Author Contributions

AV, SC-B, and HS conceived and designed the study. IA, CL, FL, FF, and MS performed the laboratory experiments. IA, CL, MF, LO, and AB carried out sample collection. VL conducted field medical monitoring. IA, AV, and HS wrote the manuscript. All authors read and approved the final manuscript.

## Funding

This study was funded by the National Council for Scientific and Technological Development (CNPQ #442522/2019-3 and #301869/2017-0), the Federal University of Pará (PAPQ/2021), and the Amazon Foundation for Studies and Support Research in Pará (FAPESPA).

## Conflict of Interest

The authors declare that the research was conducted in the absence of any commercial or financial relationships that could be construed as a potential conflict of interest.

## Publisher's Note

All claims expressed in this article are solely those of the authors and do not necessarily represent those of their affiliated organizations, or those of the publisher, the editors and the reviewers. Any product that may be evaluated in this article, or claim that may be made by its manufacturer, is not guaranteed or endorsed by the publisher.
